# Improving Google Flu Trends Estimates for the United States through Transformation

**DOI:** 10.1371/journal.pone.0109209

**Published:** 2014-12-31

**Authors:** Leah J. Martin, Biying Xu, Yutaka Yasui

**Affiliations:** 1 School of Public Health, University of Alberta, Edmonton, Alberta, Canada; 2 Zhejiang University, Hangzhou, China; Harvard School of Public Health, United States of America

## Abstract

Google Flu Trends (GFT) uses Internet search queries in an effort to provide early warning of increases in influenza-like illness (ILI). In the United States, GFT estimates the percentage of physician visits related to ILI (%ILINet) reported by the Centers for Disease Control and Prevention (CDC). However, during the 2012–13 influenza season, GFT overestimated %ILINet by an appreciable amount and estimated the peak in incidence three weeks late. Using data from 2010–14, we investigated the relationship between GFT estimates (%GFT) and %ILINet. Based on the relationship between the relative change in %GFT and the relative change in %ILINet, we transformed %GFT estimates to better correspond with %ILINet values. In 2010–13, our transformed %GFT estimates were within ±10% of %ILINet values for 17 of the 29 weeks that %ILINet was above the seasonal baseline value determined by the CDC; in contrast, the original %GFT estimates were within ±10% of %ILINet values for only two of these 29 weeks. Relative to the %ILINet peak in 2012–13, the peak in our transformed %GFT estimates was 2% lower and one week later, whereas the peak in the original %GFT estimates was 74% higher and three weeks later. The same transformation improved %GFT estimates using the recalibrated 2013 GFT model in early 2013–14. Our transformed %GFT estimates can be calculated approximately one week before %ILINet values are reported by the CDC and the transformation equation was stable over the time period investigated (2010–13). We anticipate our results will facilitate future use of GFT.

## Introduction

In the United States, traditional influenza and influenza-like illness (ILI) surveillance data are available from the Centers for Disease Control and Prevention (CDC) and include data from ILINet, an outpatient surveillance system that measures the percentage of physician visits related to ILI (%ILINet) [1]. Recently, Ginsberg *et al.* developed a model to estimate %ILINet using Internet search queries and to “provide one of the most timely, broad-reaching influenza monitoring systems available today” [2]. This model, known as Google Flu Trends (GFT), produces estimates for 29 countries [3] and has been incorporated into ILI-related research [4]–[8]. A key feature of GFT is its potential to provide early warning of increases in ILI because GFT estimates (%GFT) are available online 1–2 weeks before %ILINet is reported [2]. However, during the past five years, GFT has incorrectly estimated %ILINet twice, at critical times. In 2009, GFT did not detect the first wave of the H1N1 pandemic in the United States [9]; Google responded by recalibrating their model and subsequently more effectively estimated the second pandemic wave [9], [10]. More recently, during the “moderately severe” influenza season of 2012–13 [11], GFT overestimated the magnitude of %ILINet in the United States by an appreciable amount and estimated the peak three weeks late [9], [12]; they have since recalibrated their model for the United States for the 2013–14 season [13]. These inaccuracies in 2009 and 2012–13 have created uncertainty about GFT estimates [12] and have been attributed to changes in Internet search behaviour [10], [12] as well as to changes made by Google to its search algorithm [14]. Furthermore, the recalibrated 2013 GFT model was not made available for more than nine months after the overestimated peak in January 2013, a delay that decreases the public health value of GFT as an early warning tool for pandemic preparedness and seasonal influenza surveillance.

Given the inaccuracies and criticisms of GFT during the 2012–13 season, we investigated the relationship between GFT estimates and their intended target (%ILINet) and transformed GFT estimates to improve their estimation of %ILINet in the United States.

## Methods

We used United States national data from GFT [3] and ILI-related sentinel surveillance data (ILINet) from the CDC [15]. We based our transformation of %GFT on the data that would be available in real-time during the current week (week *i*), i.e., preliminary %ILINet (p%ILINet) values for the prior week (week *i*-1), which are the initial values reported by the CDC. We entered the p%ILINet values from the text of the weekly FluView Influenza Surveillance Reports [16]. To evaluate the prediction performance, we used final %ILINet values (f%ILINet) as the values to be predicted (the target of the prediction). There were two weeks for which p%ILINet values were not available (weeks 39 of 2009–10 and 2012–13); for these, we used the f%ILINet values.

In our main analysis for evaluating the prediction performance, we limited the data to a period after the 2009 GFT recalibration and before the 2013 GFT recalibration: i.e., week 39 of 2010 to week 30 of 2013 (October 3, 2010 to July 27, 2013; 147 weeks). Although the recalibration by Google was reported in October 2013, it was applied to the data retrospectively starting August 1, 2013 [13]; thus, our evaluation used data before August 2013. We compared the GFT estimate of %ILINet (denoted as %GFT) to %ILINet and transformed %GFT by equating it to f%ILINet*_i_* in the following equation (Equation (1)):
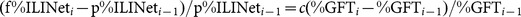



This equation states that the relative change in %ILINet from the previous week is proportional to the relative change in %GFT from the previous week; in practice, the former is the relative change from the preliminary %ILINet value (i.e., the predictor) available from the previous week (week *i*-1) to the final %ILINet value (i.e., the target) for the current week (week *i*). We estimated *c* by least squares regression (i.e., we set the relative change from the previous week in %GFT*_i_* = x and that in %ILINet*_i_* = y and created a simple linear regression model without an intercept).

In addition to our main analysis, we conducted four subanalyses. First, we assessed whether the same transformation parameter (*c*) developed for the 2010–13 data would be useful for the recalibrated 2013 GFT model by testing our transformation equation with the same parameter value for *c* on the most recent data available at the time of analysis using this model (weeks 31 to 10 of the 2013–14 influenza season: July 28, 2013 to March 8, 2014; 32 weeks). Second, we calculated a separate value of *c* based on weeks 31 to 10 of the 2013–14 influenza season alone. Third, we assessed whether a similar transformation could have been developed *before* the large overestimation in GFT that occurred during the 2012–13 season using data for week 39 of 2010 to week 39 of 2012. Fourth, as a supplemental analysis, we reran the transformation separately for each of the ten United States Department of Health and Human Services (HHS) regions to examine how the value of *c* varied across the country. We used the final regional %ILINet values in these regional calculations as the historic preliminary regional values are not made available by CDC. The latter three subanalyses used the same methodology to estimate *c* as our main transformation analysis.

We compared our transformed GFT estimates to the %ILINet value using four metrics: 1) the proportion of weeks in which the relative percent difference between the transformed %GFT estimate and the f%ILINet value was within ±5% or ±10% of the f%ILINet value, 2) sum of the squared errors, 3) relative percentage difference in peak magnitude in 2012–13, and 4) difference in peak timing in 2012–13. We limited our evaluation of (1) to weeks when f%ILINet was above the CDC’s reported national baseline value for that season: 2.5% for 2010–11 [17], 2.4% for 2011–12 [18], 2.2% for 2012–13 [19], and 2.0% for 2013–14 [20]. For comparison, we also calculated these four metrics for the original GFT estimates and the previous week’s p%ILINet value (for metrics 1 and 2 only). We made these comparisons for 2010–13 and 2013–14 separately. In addition, as a supplementary analysis, we made these comparisons for estimates from two models reported by Lazer *et al*., who developed regression models to improve upon GFT estimates [14].

Ethics approval was not required because all data were publicly available. Analyses were conducted using SAS 9.4 (SAS Institute Inc., Cary, North Carolina) and R version 3.0.2 [21]. To make these transformed GFT estimates available to researchers and public health professionals and testable on future GFT and ILINet data, we provide the coding and the data we created [22].

## Results

**Figure 1:**
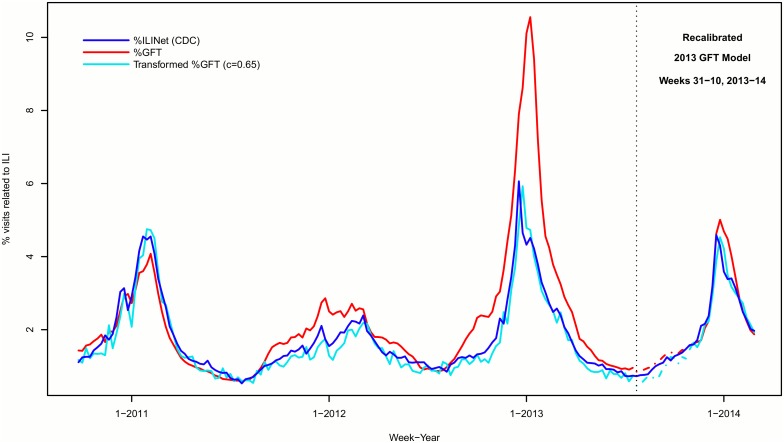
Weekly percentage of sentinel physician visits related to influenza-like illness (ILI) reported by the Centers for Disease Control and Prevention (CDC) and estimated using Google Flu Trends (GFT), United States, October 2010–March 2014. The final CDC value (f%ILINet; blue) is compared to the GFT estimate (%GFT; red) and the transformed GFT estimate using *c* = 0.65 (transformed %GFT; turquoise). The GFT model was recalibrated during the 2013–14 season: dashed lines show the period in which GFT estimates were retrospectively re-estimated using the 2013 GFT model.

We found that the relative change in %GFT closely approximates the relative change from p%ILINet to f%ILINet and this relationship can inform %GFT for the current week, *i*, to better match its target, f%ILINet in week *i*. Specifically, we identified that the relative change in %ILINet from its preliminary value in week *i*-1 to its final value in week *i* is 0.65 times the relative change in %GFT from week *i*-1 to week *i*. Using this relationship, we transformed %GFT in week *i* (%GFT*_i_*) so that it better corresponds with f%ILINet*_i_* by equating it with f%ILINet*_i_* in rearranged Equation (1):
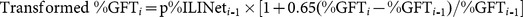



This transformation produces GFT estimates that more closely approximate %ILINet values compared to the original %GFT estimates (Fig. 1). In 2012–13, the transformed GFT estimates peaked at 5.9%, a relative difference of −2.2% compared to the %ILINet peak of 6.1%. Transformation shifted the peak in %GFT two weeks earlier, from week 3 to week 1, which was one week after the peak in %ILINet (week 52) (Fig. 1). The sum of the squared errors between %ILINet*_i_* and the transformed %GFT*_i_* was 12.1; however, by using p%ILINet*_i_*_-1_ on its own as an estimate of f%ILINet*_i_*, the sum of the squared errors (i.e., (f%ILINet*_i_*-p%ILINet*_i_*_-1_)^2^) was appreciably larger (17.0). This suggests that %GFT*_i_* provides useful information about the final %ILINet*_i_* beyond the preliminary %ILINet value from the preceding week. In comparison, the sum of the squared errors for the original %GFT estimate was 177.4 (Table 1). Our transformed %GFT estimate was within ±5% and ±10% of %ILINet 28% and 59%, respectively, of the 29 weeks in 2010–13 when %ILINet was above baseline, which was equal to or more frequent than the other estimates we compared (Table 1). For example, during these 29 weeks, the 2009 GFT estimate was never within ±5% of %ILINet and only within ±10% of %ILINet 7% of the above baseline weeks (Table 1).

**Table 1:** Comparing estimates of the weekly percentage of physician visits related to influenza-like illness (ILI) based on Google Flu Trends (GFT) to values reported by the Centers for Disease Control and Prevention (CDC), United States, October 2010–March 2014.

Estimate	2010–13 seasons(Week 40, 2010 to Week 30, 2013)*					2013–14 season(Week 31, 2013 to Week 10, 2014)*				
	No. (%)above baseline weeks** within ±5%	No. (%)above baselineweeks within ±10%	Sum of squared errors	Relative % difference in peak magnitude (2012–13)	Difference in peak timing (2012–13)	No. (%)above baselineweeks† within ±5%	No. (%)above baselineweeks within ±10%	Sum of squared errors	Relative % difference in peak magnitude	Difference in peak timing
Original2009%GFT	0 (0.0)	2 (6.9)	177.4	74.1	3 weeks after	-	-	-	-	-
Recalibrated 2013%GFT	-	-	-	-	-	6 (43)	8 (57)	3.8	9.1	1 week after
Preliminary %ILINet from previous week	8 (28)	12 (41)	17.0	-	-	2 (14)	5 (36)	5.7	-	-
Transformed%GFT (*c* = 0.65)	8 (28)	17 (59)	12.1	–2.2	1 week after	5 (36)	11 (79)	2.1	−1.3	1 week after
Transformed %GFT (*c* = 1.1)	-	-	-	-	-	6 (44)	10 (71)	1.6	2.4	1 week after

*Week 39 of 2010 and week 30 of 2013 were used to calculate transformed %GFT estimates for each of these time periods, respectively.

**During the 2010–13 seasons, 29 weeks were above baseline.

† During the 2013–14 season, 14 weeks were above baseline.

In our first subanalysis, when we applied the transformation using *c* = 0.65 to data from the 2013–14 season, we see similar results as our main analysis, with the transformed %GFT estimates corresponding well with the %ILINet values (Fig. 1). In our second subanalysis, when we reran the least square estimation using only the data from weeks 31–10 of the 2013–14 season, the value of *c* changed somewhat, increasing to 1.1, which created some changes to our results for these weeks, including increasing the peak magnitude (Table 1; S1 Fig.). In our third subanalysis, using data from 2010–12 only, the value of *c* determined in the regression of the relative change in %GFT (x) on the relative change in %ILINet (y) was 0.66, almost identical to the estimate in our main analysis for 2010–13 (*c = *0.65). In our fourth subanalysis, our regional analyses, we found values of *c* to range from 0.35 in HHS region 1 to 1.0 in HHS region 7 (S2-S11 Figs.; S1 Table). In our supplementary analysis, we found that our transformed %GFT estimates were closer to %ILINet values than the two main models reported by Lazer *et al*. (S12 Fig.; S2 Table).

## Discussion

We transformed the percentage of sentinel physician visits related to ILI estimated by GFT to better match the ILINet value reported by the CDC, thus reducing the overestimation produced by GFT during the 2012–13 influenza season by an absolute difference of 4.4 percentage points and shifting the peak ahead by two weeks. Relative to the peak in %ILINet, the transformed GFT peak in 2012–13 was only 2.2% lower in magnitude (an absolute difference of 0.13 percentage points) and one week later. Key features of this transformation are that it can be calculated approximately one week before the final %ILINet value is reported by the CDC and it appears stable over the time period investigated (2010–13).

The stability of our transformation equation is important because it means that the same equation with the same value of *c* could be applied continuously from 2010–13. For example, we found that, using data from 2010–12, transformed GFT estimates could have been calculated using this equation before the 2012–13 season, which would have corrected for the vast overestimation by GFT before it occurred. Furthermore, we found that this equation also appears to work during the beginning of the 2013–14 season, improving upon the recalibrated 2013 GFT model. However, we need additional prospectively estimated values from this newly recalibrated 2013 GFT model to draw more definitive conclusions. Assuming continued stability, this relationship suggests that the value of *c* and the associated transformed GFT estimates do not need to be recalculated at the beginning of each influenza season, which is a critical period of time to monitor. Despite this observed stability, continued monitoring and evaluation of GFT estimates, our transformed estimates, and their relationship with ILI surveillance data from the CDC are needed.

Although our transformed GFT estimate closely matches %ILINet, the largest discrepancy between these values (1.3 percentage points) was seen during week 1 in 2012–13, when %ILINet peaked in week 52 and our transformed %GFT peaked in week 1. However, the height of the peak in %ILINet may have been somewhat artifactual due to a decrease in total physician visits (the denominator of %ILINet) during the Christmas holidays in week 52. In contrast, the absolute number of ILI-related physician visits (the numerator of %ILINet) peaked in week 2 of 2012–13, one week after our transformed %GFT estimate peaked. We observed the same phenomenon in our subanalysis of 2013–14, when %ILINet peaked in week 52 but its numerator peaked in week 1, the same week as our transformed %GFT estimate peaked.

We note several limitations to our study. First, our main analysis was conducted using the previous 2009 GFT model, which Google recalibrated in October 2013. Although our transformation appears to work in the first 32 weeks of the recalibrated 2013 GFT model, continued evaluation and monitoring are needed; our transformation needs to be more thoroughly assessed and potentially modified using the new 2013 GFT model and any future GFT models, which may have different relationships with %ILINet. Additionally, the estimates reported by GFT for August-October 2013 were not prospectively estimated; rather, they were retrospectively updated with the recalibrated model. Second, our approach to transform GFT estimates using the most recent %ILINet value would create somewhat of a delay in reporting GFT estimates. Given that the p%ILINet value for week *i*-1 is available on Friday of week *i* and the %GFT*_i_* estimate is updated until Saturday of week *i*, we could conduct the transformation on Friday or Saturday of week *i*, approximately one week before p%ILINet*_i_* is released. This is a distinct advantage in timing over %ILINet. Third, Google has not made available the search terms used in their GFT models [2]; therefore, as others have emphasized, we are limited in our ability to investigate and understand GFT estimates and their relationship with %ILINet [9], [14]. One strategy researchers have used is to develop their own models based on search terms of their choosing, for example, using Google Trends [23]–[25]. Recently, Santillana *et al.* developed an “alternative methodology” to GFT using Google Correlate to improve upon %GFT estimates [26]. Our approach, which can be viewed as complementary to these investigations, may provide insights for users of GFT as well as researchers developing their own digital disease surveillance models.

Herein we provide a simple transformation that can be implemented prospectively to improve GFT estimates for the United States, approximately one week before %ILINet values are reported. We anticipate our results could help to inform future use of GFT.

## Supporting Information

S1 Fig **Weekly percentage of sentinel physician visits related to influenza-like illness (ILI) reported by the Centers for Disease Control and Prevention (CDC) and estimated using Google Flu Trends (GFT), United States, 2013–14.** The CDC value (%ILINet; blue) is compared to the transformed GFT estimate using *c* = 0.65 (transformed %GFT; turquoise) and using *c* = 1.1 (transformed %GFT; green). For reference, we show the GFT estimate using the recalibrated 2013 GFT model (recalibrated 2013%GFT; red). The dashed lines show the period of time when the recalibrated 2013 GFT model was applied retrospectively from week 44 back to week 31.
(TIF)

S2 Fig **Weekly percentage of sentinel physician visits related to influenza-like illness (ILI) reported by the Centers for Disease Control and Prevention (CDC) and estimated using Google Flu Trends (GFT), HHS Region 1, October 2010–July 2013.** The CDC value (%ILINet; blue) is compared to the original GFT estimate (%GFT; red), and the transformed GFT estimate (transformed %GFT; turquoise).
(TIF)

S3 Fig **Weekly percentage of sentinel physician visits related to influenza-like illness (ILI) reported by the Centers for Disease Control and Prevention (CDC) and estimated using Google Flu Trends (GFT), HHS Region 2, October 2010–July 2013.** The CDC value (%ILINet; blue) is compared to the original GFT estimate (%GFT; red), and the transformed GFT estimate (transformed %GFT; turquoise).
(TIF)

S4 Fig **Weekly percentage of sentinel physician visits related to influenza-like illness (ILI) reported by the Centers for Disease Control and Prevention (CDC) and estimated using Google Flu Trends (GFT), HHS Region 3, October 2010–July 2013.** The CDC value (%ILINet; blue) is compared to the original GFT estimate (%GFT; red), and the transformed GFT estimate (transformed %GFT; turquoise).
(TIF)

S5 Fig **Weekly percentage of sentinel physician visits related to influenza-like illness (ILI) reported by the Centers for Disease Control and Prevention (CDC) and estimated using Google Flu Trends (GFT), HHS Region 4, October 2010–July 2013.** The CDC value (%ILINet; blue) is compared to the original GFT estimate (%GFT; red), and the transformed GFT estimate (transformed %GFT; turquoise).
(TIF)

S6 Fig **Weekly percentage of sentinel physician visits related to influenza-like illness (ILI) reported by the Centers for Disease Control and Prevention (CDC) and estimated using Google Flu Trends (GFT), HHS Region 5, October 2010–July 2013.** The CDC value (%ILINet; blue) is compared to the original GFT estimate (%GFT; red), and the transformed GFT estimate (transformed %GFT; turquoise).
(TIF)

S7 Fig **Weekly percentage of sentinel physician visits related to influenza-like illness (ILI) reported by the Centers for Disease Control and Prevention (CDC) and estimated using Google Flu Trends (GFT), HHS Region 6, October 2010–July 2013.** The CDC value (%ILINet; blue) is compared to the original GFT estimate (%GFT; red), and the transformed GFT estimate (transformed %GFT; turquoise).
(TIF)

S8 Fig **Weekly percentage of sentinel physician visits related to influenza-like illness (ILI) reported by the Centers for Disease Control and Prevention (CDC) and estimated using Google Flu Trends (GFT), HHS Region 7, October 2010–July 2013.** The CDC value (%ILINet; blue) is compared to the original GFT estimate (%GFT; red), and the transformed GFT estimate (transformed %GFT; turquoise).
(TIF)

S9 Fig **Weekly percentage of sentinel physician visits related to influenza-like illness (ILI) reported by the Centers for Disease Control and Prevention (CDC) and estimated using Google Flu Trends (GFT), HHS Region 8, October 2010–July 2013.** The CDC value (%ILINet; blue) is compared to the original GFT estimate (%GFT; red), and the transformed GFT estimate (transformed %GFT; turquoise).
(TIF)

S10 Fig **Weekly percentage of sentinel physician visits related to influenza-like illness (ILI) reported by the Centers for Disease Control and Prevention (CDC) and estimated using Google Flu Trends (GFT), HHS Region 9, October 2010–July 2013.** The CDC value (%ILINet; blue) is compared to the original GFT estimate (%GFT; red), and the transformed GFT estimate (transformed %GFT; turquoise).
(TIF)

S11 Fig **Weekly percentage of sentinel physician visits related to influenza-like illness (ILI) reported by the Centers for Disease Control and Prevention (CDC) and estimated using Google Flu Trends (GFT), HHS Region 10, October 2010–July 2013.** The CDC value (%ILINet; blue) is compared to the original GFT estimate (%GFT; red), and the transformed GFT estimate (transformed %GFT; turquoise).
(TIF)

S12 Fig **Weekly percentage of physician visits related to influenza-like illness (ILI) reported by the Centers for Disease Control and Prevention (CDC) and estimated using Google Flu Trends (GFT), United States, 2010–13.** The CDC value (%ILINet; blue) is compared to the transformed GFT estimate (transformed %GFT; turquoise) and estimates from Lazer *et al.* using (A) lagged %ILINet values (green) and (B) GFT estimates combined with %ILINet values (grey). For reference, we also show the GFT estimate (%GFT; red).
(TIF)

S1 Table **Comparing values of** ***c*** **for the ten Health and Human Services (HHS) regions, United States, October 2010–July 2013.**
(DOCX)

S2 Table **Comparing estimates of the weekly percentage of physician visits related to influenza-like illness (ILI) based on Google Flu Trends (GFT) to values reported by the Centers for Disease Control and Prevention (CDC), United States, October 2010–July 2013.**
(DOCX)
