# Correction: Improving Google Flu Trends Estimates for the United States through Transformation

**DOI:** 10.1371/journal.pone.0122939

**Published:** 2015-04-21

**Authors:** Leah J. Martin, Biying Xu, Yutaka Yasui

Reference 27 is omitted from the last sentence of the second last paragraph of the Methods section. The sentence should read: In addition, as a supplementary analysis, we made these comparisons for estimates from two models reported by Lazer et al., who developed regression models to improve upon GFT estimates [14, 27].

The reference is: Lazer D, Kennedy R, King G, Vespignani A (2014) Replication data for: The parable of Google Flu: Traps in big data analysis. Available: http://dx.doi.org/10.7910/DVN/24823 UNF:5:BJh9WzZQNEeSEpV3EWs+xg== IQSS Dataverse Network [Distributor] V1 [Version].

In the "Estimate" column of S2 Table, reference 21 should be replaced with reference 27. Please view the correct S2 Table below.

## Supporting Information

S2 Table Comparing estimates of the weekly percentage of physician visits related to influenza-like illness (ILI) based on Google Flu Trends (GFT) to values reported by the Centers for Disease Control and Prevention (CDC), United States, October 2010-July 2013. (DOCX)
