# Supplementary material for: Improving Google Flu Trends Estimates for the United States through Transformation
Source: PLoS One. 2014 Dec 31;9(12):e109209. doi: 10.1371/journal.pone.0109209 (PMC4281210; doi:10.1371/journal.pone.0109209)
Supplement: S1 Table — Comparing values of c for the ten Health and Human Services (HHS) regions, United States, October 2010–July 2013. (DOCX) [file pone.0109209.s013.docx]

**Table S1. Comparing values of *c* for the ten Health and Human Services (HHS) regions, United States, October 2010 – July 2013**

| HHS Region | | *c* |
| --- | --- | --- |
| 1 | Connecticut, Maine, Massachusetts, New Hampshire, Rhode Island, and Vermont | 0.35 |
| 2 | New Jersey, New York, Puerto Rico, and the Virgin Islands* | 0.60 |
| 3 | Delaware, District of Columbia, Maryland, Pennsylvania, Virginia, and West Virginia | 0.70 |
| 4 | Alabama, Florida, Georgia, Kentucky, Mississippi, North Carolina, South Carolina, and Tennessee | 0.64 |
| 5 | Illinois, Indiana, Michigan, Minnesota, Ohio, and Wisconsin | 0.68 |
| 6 | Arkansas, Louisiana, New Mexico, Oklahoma, and Texas | 0.63 |
| 7 | Iowa, Kansas, Missouri, and Nebraska | 1.0 |
| 8 | Colorado, Montana, North Dakota, South Dakota, Utah, and Wyoming | 0.56 |
| 9 | Arizona, California, Hawaii, Nevada, American Samoa, Commonwealth of the Northern Mariana Islands, Federated States of Micronesia, Guam, Marshall Islands, and Republic of Palau** | 0.40 |
| 10 | Alaska, Idaho, Oregon, and Washington | 0.75 |

*For HHS Region 2, GFT only lists NJ and NY

**For HHS Region 9, GFT only lists AZ, CA, HI, and NV

Note: *c* was calculated using Equation 1 and the final %ILINet values and %GFT estimates for each HHS region: preliminary regional %ILINet values are not available. Therefore, these regional relationships may have differed if constructed using the data available in real-time.
