# Supplementary material for: Improving Google Flu Trends Estimates for the United States through Transformation
Source: PLoS One. 2014 Dec 31;9(12):e109209. doi: 10.1371/journal.pone.0109209 (PMC4281210; doi:10.1371/journal.pone.0109209)
Supplement: S2 Table — Comparing estimates of the weekly percentage of physician visits related to influenza-like illness (ILI) based on Google Flu Trends (GFT) to values reported by the Centers for Disease Control and Prevention (CDC), United States, October 2010–July 2013. (DOCX) [file pone.0109209.s014.docx]

**Table S2. Comparing estimates of the weekly percentage of physician visits related to influenza-like illness (ILI) based on Google Flu Trends (GFT) to values reported by the Centers for Disease Control and Prevention (CDC), United States, October 2010 – July 2013.**

| **Estimate** | **2010-13 seasons**  **(Week 40, 2010 to Week 30, 2013)*** | | | | |
| --- | --- | --- | --- | --- | --- |
|  | **No. (%)**  **above baseline weeks** within ±5% of %ILINet** | **No. (%)**  **above baseline weeks within ±10% of %ILINet** | **Sum of Squared Errors** | **Relative % difference in peak magnitude**  **(2012-13)** | **Difference in peak timing (2012-13)** |
| Lazer *et al.*  Equation 6 (“dlscflu09”) [[14](#_ENREF_14),21] | 4 (14) | 10 (34) | 31.2 | 15 | 2 weeks after |
| Lazer *et al.*  Equation 9 (“degflu09”) [[14](#_ENREF_14),21] | 7 (24) | 13 (45) | 36.8 | 30 | 2 weeks after |
| Transformed GFT  (*c*=0.65) | 8 (28) | 17 (59) | 12.1 | -2.2 | 1 week after |

*Week 39 of 2010 was used in calculations to determine the transformed %GFT values

**During the 2010-13 season, 29 weeks were above baseline
