# Supplementary material for: Correction: Improving Google Flu Trends Estimates for the United States through Transformation
Source: PLoS One. 2015 Apr 21;10(4):e0122939. doi: 10.1371/journal.pone.0122939 (PMC4405189; doi:10.1371/journal.pone.0122939)
Supplement: S2 Table — (DOCX) [file pone.0122939.s001.docx]

**Table S2. Comparing estimates of the weekly percentage of physician visits related to influenza-like illness (ILI) based on Google Flu Trends (GFT) to values reported by the Centers for Disease Control and Prevention (CDC), United States, October 2010 – July 2013.**

| **Estimate** | **2010-13 seasons**  **(Week 40, 2010 to Week 30, 2013)*** | | | | |
| --- | --- | --- | --- | --- | --- |
|  | **No. (%)**  **above baseline weeks** within ±5% of %ILINet** | **No. (%)**  **above baseline weeks within ±10% of %ILINet** | **Sum of Squared Errors** | **Relative % difference in peak magnitude**  **(2012-13)** | **Difference in peak timing (2012-13)** |
| Lazer *et al.*  Equation 6 (“dlscflu09”) [[14](#_ENREF_14),27] | 4 (14) | 10 (34) | 31.2 | 15 | 2 weeks after |
| Lazer *et al.*  Equation 9 (“degflu09”) [[14](#_ENREF_14),27] | 7 (24) | 13 (45) | 36.8 | 30 | 2 weeks after |
| Transformed GFT  (*c*=0.65) | 8 (28) | 17 (59) | 12.1 | -2.2 | 1 week after |

*Week 39 of 2010 was used in calculations to determine the transformed %GFT values

**During the 2010-13 season, 29 weeks were above baseline
